# Supplementary material for: The Effect of Semaglutide on Blood Pressure in Patients without Diabetes: A Systematic Review and Meta-Analysis
Source: J Clin Med. 2023 Jan 18;12(3):772. doi: 10.3390/jcm12030772 (PMC9917722; doi:10.3390/jcm12030772)
Supplement: Supplementary file 1 [file jcm-12-00772-s001.zip › jcm-2122074-supplementary.pdf]

Supplementary Material

Search strategy

Publication type	RCT Medline/Embase:<br>1. 'semaglutide'/exp<br>2. (ozempic OR rybelsus OR semaglutide OR wegovy):ti,ab,kw<br>3. #1 OR #2<br>4. 'abnormal blood pressure'/exp OR 'blood pressure'/exp OR 'hypotension'/exp OR 'hypertension'/exp OR 'blood pressure monitoring'/exp<br>5. ('Blood pressure' OR hypertensi* OR hypotensi*):ti,ab,kw<br>6. #4 OR #5<br>7. 'clinical trial'/de OR 'randomized controlled trial'/de OR 'randomization'/de OR 'single blind procedure'/de OR 'double blind procedure'/de OR 'crossover procedure'/de OR 'placebo'/de OR 'prospective study'/de OR ('randomi?ed controlled' NEXT/1 trial*) OR rct OR 'randomly allocated' OR 'allocated randomly' OR 'random allocation' OR (allocated NEAR/2 random) OR (single NEXT/1 blind*) OR (double NEXT/1 blind*) OR ((treble OR triple) NEAR/1 blind*) OR placebo*<br>8. #3 AND #6 AND #7
Geography/Location	No restriction
Publication date	From inception of database until July 1st 2022
Language	No restriction
Age	No restriction

Cochrane search

Date Run: 18/07/2022

(ozempic OR rybelsus OR semaglutide OR wegovy):ti,ab,kw

[mh "Blood Pressure Determination"] OR [mh "Blood Pressur"] OR [mh "Hypotension"] OR [mh "Hypertension"]

("Blood pressure" OR hypertensi* OR hypotensi*):ti,ab,kw

#2 OR #3

#1 AND #4

Medline (Ovid) search

Date Run: 18/07/2022

Randomized controlled trials as Topic/

Randomized controlled trial/

Random allocation/

Double blind method/

Single blind method/

Clinical trial/

exp Clinical Trials as Topic/

or/1-7

(clinic$ adj trial$1).tw.

((singl$ or doubl$ or treb$ or tripl$) adj (blind$3 or mask$3)).tw.

Placebos/

Placebo$.tw.

Randomly allocated.tw.

(allocated adj2 random).tw.

or/9-14

8 or 15

Case report.tw.

Letter/

Historical article/

Review of reported cases.pt.

Review, multicase.pt.

or/17-21

16 not 22

(ozempic OR rybelsus OR semaglutide OR wegovy).ti,ab.

Blood Pressure Determination/ OR exp Blood Pressure/ OR exp Hypotension/ OR exp Hypertension/

(Blood pressure or hypertensi* or hypotensi*).ti,ab.

25 OR 26

23 AND 24 AND 27

CINAHL

Date Run: 18/07/2022

TI (ozempic OR rybelsus OR semaglutide OR wegovy) OR AB (ozempic OR rybelsus OR semaglutide OR wegovy)

(MH "Blood Pressure+") OR (MH "Blood Pressure Determination") OR (MH "Hypertension+") OR (MH "Hypotension+")

TI ("Blood pressure" OR hypertensi* OR hypotensi*) OR AB ("Blood pressure" OR hypertensi* OR hypotensi*)

S2 OR S3

TX allocat* random* OR (MH "Quantitative Studies") OR (MH "Placebos") OR TX placebo* OR TX random* allocat* OR (MH "Random Assignment") OR TX randomi* control* trial* OR TX ((singl* n1 blind*) OR (singl* n1 mask*)) OR TX ((doubl* n1 blind*) OR (doubl* n1 mask*)) OR TX ((tripl* n1 blind*) OR (tripl* n1 mask*)) OR TX ((trebl* n1 blind*) OR (trebl* n1 mask*)) OR TX clinic* n1 trial* OR PT Clinical trial OR (MH "Clinical Trials+")

S1 AND S4 AND S5

Web of Science

(ozempic OR rybelsus OR semaglutide OR wegovy) AND ("blood pressure" OR hypotensi* OR hypertensi*) AND (randomised OR randomized OR randomisation OR randomisation OR placebo* OR (random* AND (allocat* OR assign*)) OR (blind* AND (single OR double OR treble OR triple)))

Google Scholar

Ozempic|rybelsus|semaglutide|wegovy "blood pressure"|hypotension|hypertension

randomised|randomized|randomisation|randomisation|placebo

Figure S1: Leave-one-out sensitivity analysis of the treatment effect on SBP.

Systolic Blood Pressure

Study name	Statistics with study removed						
	Point	Standard error	Variance	Lower limit	Upper limit	Z-Value	p-Value
O'Neil, 0.05mg	-4,878	0,420	0,177	-5,702	-4,054	-11,601	0,000
O'Neil,0.1mg	-4,848	0,420	0,177	-5,672	-4,024	-11,531	0,000
O'Neil, 0.2mg	-4,836	0,421	0,177	-5,661	-4,012	-11,501	0,000
O'Neil, 0.3mg	-4,833	0,421	0,177	-5,657	-4,009	-11,492	0,000
O'Neil, 0.4mg	-4,847	0,421	0,177	-5,671	-4,023	-11,525	0,000
O'Neil, 0.3mg FE	-4,840	0,420	0,177	-5,664	-4,017	-11,516	0,000
O'Neil, 0.4mg FE	-4,742	0,421	0,177	-5,567	-3,918	-11,277	0,000
STEP 1, 2.4mg	-4,627	0,553	0,306	-5,711	-3,543	-8,365	0,000
STEP 3, 2.4mg	-4,936	0,441	0,194	-5,800	-4,072	-11,199	0,000
STEP 4, 2.4mg	-5,042	0,460	0,212	-5,944	-4,141	-10,961	0,000
STEP 5, 2.4mg	-4,884	0,430	0,185	-5,727	-4,041	-11,359	0,000
STEP 8, 2.4mg	-4,633	0,426	0,181	-5,468	-3,799	-10,883	0,000
	-4,833	0,416	0,173	-5,647	-4,018	-11,626	0,000

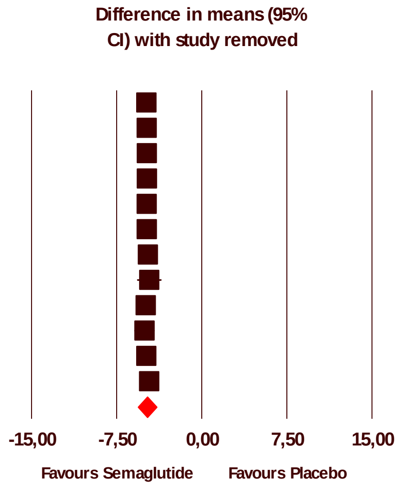

Figure S2: Leave-one-out sensitivity analysis of the treatment effect on DBP.

Diastolic Blood Pressure

Study name	Statistics with study removed						
	Point	Standard error	Variance	Lower limit	Upper limit	Z-Value	p-Value
O'Neil, 0.05mg	-2,529	0,639	0,408	-3,781	-1,277	-3,959	0,000
O'Neil,0.1mg	-2,523	0,639	0,408	-3,776	-1,271	-3,948	0,000
O'Neil, 0.2mg	-2,434	0,639	0,408	-3,685	-1,182	-3,811	0,000
O'Neil, 0.3mg	-2,503	0,639	0,409	-3,756	-1,250	-3,915	0,000
O'Neil, 0.4mg	-2,464	0,640	0,409	-3,718	-1,210	-3,852	0,000
O'Neil, 0.3mg FE	-2,550	0,638	0,407	-3,801	-1,299	-3,995	0,000
O'Neil, 0.4mg FE	-2,344	0,635	0,403	-3,589	-1,100	-3,693	0,000
STEP 1, 2.4mg	-2,418	0,736	0,542	-3,861	-0,975	-3,284	0,001
STEP 3, 2.4mg	-2,465	0,672	0,452	-3,783	-1,147	-3,666	0,000
STEP 4, 2.4mg	-2,717	0,565	0,320	-3,825	-1,609	-4,806	0,000
STEP 5, 2.4mg	-2,334	0,646	0,418	-3,601	-1,067	-3,611	0,000
STEP 8, 2.4mg	-1,823	0,283	0,080	-2,377	-1,269	-6,449	0,000
	-2,445	0,614	0,377	-3,648	-1,242	-3,984	0,000

Difference in means (95% CI) with study removed

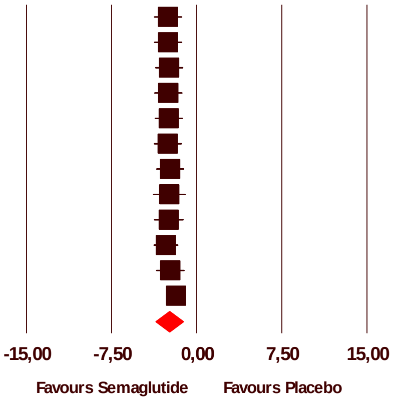

Figure S3: Forest plot of semaglutide vs placebo showing the pooled weighted mean difference for SBP (fixed effect model).

Systolic Blood Pressure

Study name	Difference in means	Standard error	Variance	Lower limit	Upper limit	Z-Value	p-Value
O'Neil, 0.05mg	-2,880	2,769	7,670	-8,308	2,548	-1,040	0,298
O'Neil,0.1mg	-4,180	2,779	7,723	-9,627	1,267	-1,504	0,133
O'Neil, 0.2mg	-4,680	2,747	7,548	-10,065	0,705	-1,703	0,088
O'Neil, 0.3mg	-4,830	2,747	7,548	-10,215	0,555	-1,758	0,079
O'Neil, 0.4mg	-4,230	2,734	7,473	-9,588	1,128	-1,547	0,122
O'Neil, 0.3mg FE	-4,490	2,802	7,850	-9,981	1,001	-1,603	0,109
O'Neil, 0.4mg FE	-8,680	2,745	7,533	-14,059	-3,301	-3,163	0,002
STEP 1, 2.4mg	-5,100	0,630	0,397	-6,335	-3,865	-8,094	0,000
STEP 3, 2.4mg	-4,000	1,250	1,562	-6,450	-1,550	-3,200	0,001
STEP 4, 2.4mg	-3,900	0,971	0,942	-5,802	-1,998	-4,018	0,000
STEP 5, 2.4mg	-4,100	1,628	2,649	-7,290	-0,910	-2,519	0,012
STEP 8, 2.4mg	-8,900	1,923	3,698	-12,669	-5,131	-4,628	0,000
	-4,833	0,416	0,173	-5,647	-4,018	-11,626	0,000

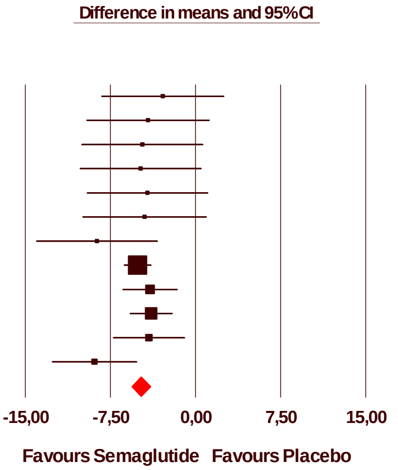

Figure S4: Forest plot of semaglutide vs placebo showing the pooled weighted mean difference for DBP (fixed effect model).

Diastolic Blood Pressure

Study name	Statistics for each study						
	Difference in means	Standard error	Variance	Lower limit	Upper limit	Z-Value	p-Value
O'Neil, 0.05mg	-1,050	1,939	3,758	-4,849	2,749	-0,542	0,588
O'Neil,0.1mg	-1,150	1,931	3,730	-4,935	2,635	-0,595	0,552
O'Neil, 0.2mg	-2,590	1,916	3,671	-6,345	1,165	-1,352	0,176
O'Neil, 0.3mg	-1,480	1,916	3,671	-5,235	2,275	-0,772	0,440
O'Neil, 0.4mg	-2,110	1,885	3,555	-5,805	1,585	-1,119	0,263
O'Neil, 0.3mg FE	-0,700	1,954	3,819	-4,530	3,130	-0,358	0,720
O'Neil, 0.4mg FE	-4,020	1,894	3,588	-7,732	-0,308	-2,122	0,034
STEP 1, 2.4mg	-2,410	0,449	0,202	-3,290	-1,530	-5,368	0,000
STEP 3, 2.4mg	-2,200	0,842	0,709	-3,850	-0,550	-2,613	0,009
STEP 4, 2.4mg	-1,300	0,098	0,010	-1,492	-1,108	-13,291	0,000
STEP 5, 2.4mg	-3,600	1,273	1,620	-6,095	-1,105	-2,828	0,005
STEP 8, 2.4mg	-4,900	0,393	0,155	-5,670	-4,130	-12,465	0,000
	-1,572	0,091	0,008	-1,751	-1,393	-17,213	0,000

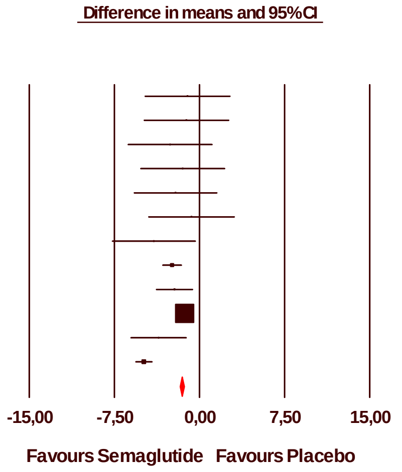

Figure S5: Funnel plot of the effect of semaglutide on SBP.

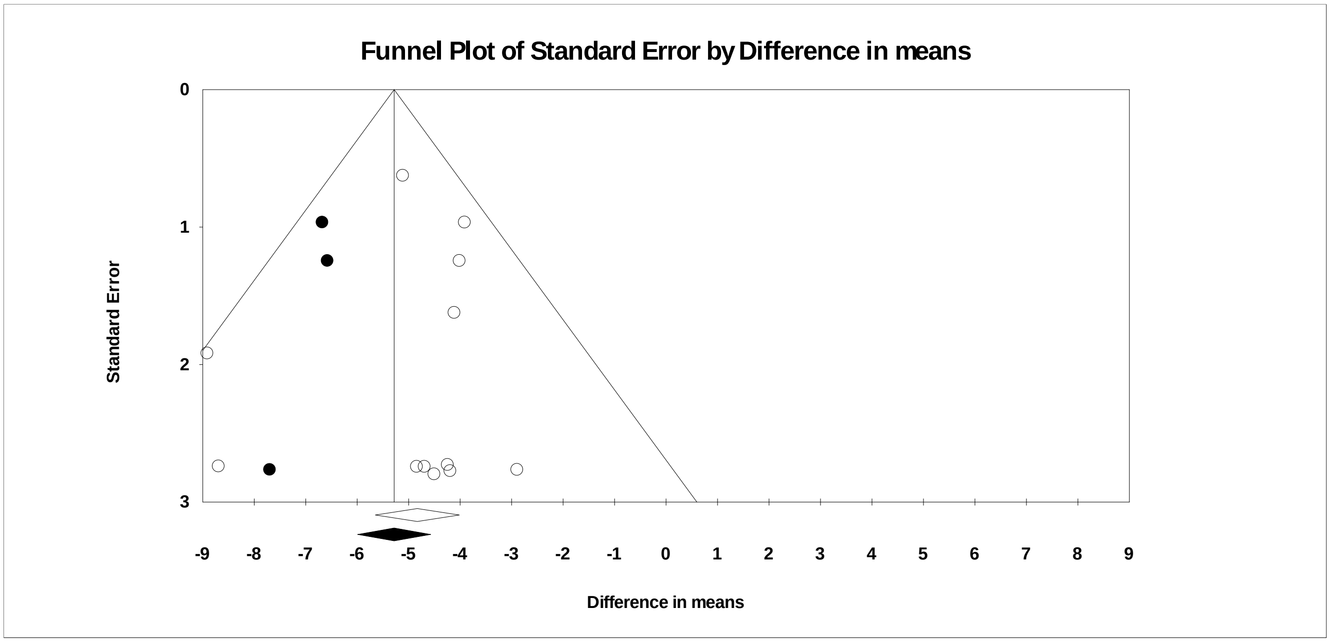

Figure S6: Meta-regression analysis of the duration of follow-up and effect of semaglutide on SBP (mmHg). The duration of follow-up is in weeks.

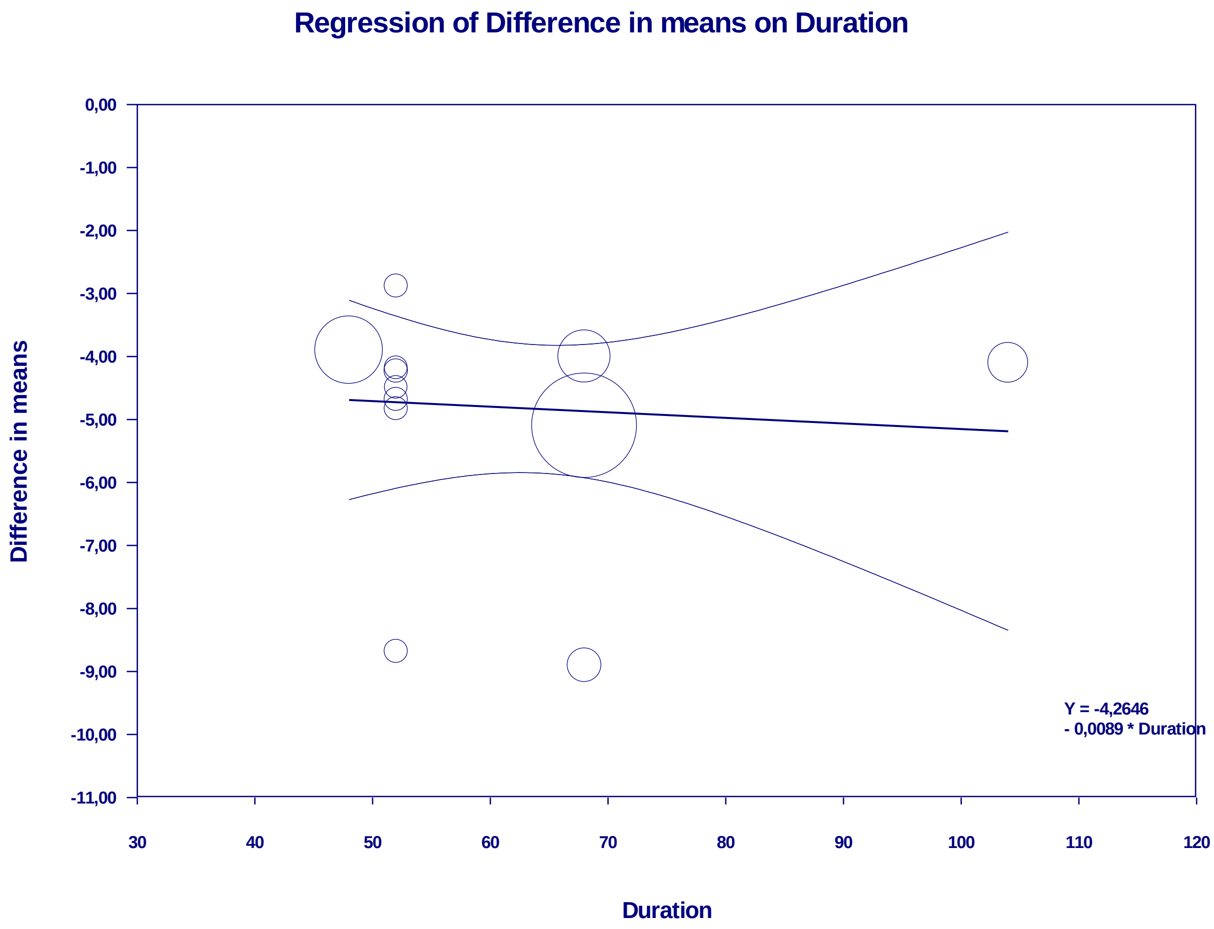

Figure S7: Meta-regression analysis of the semaglutide dose (mg) on SBP (mmHg).

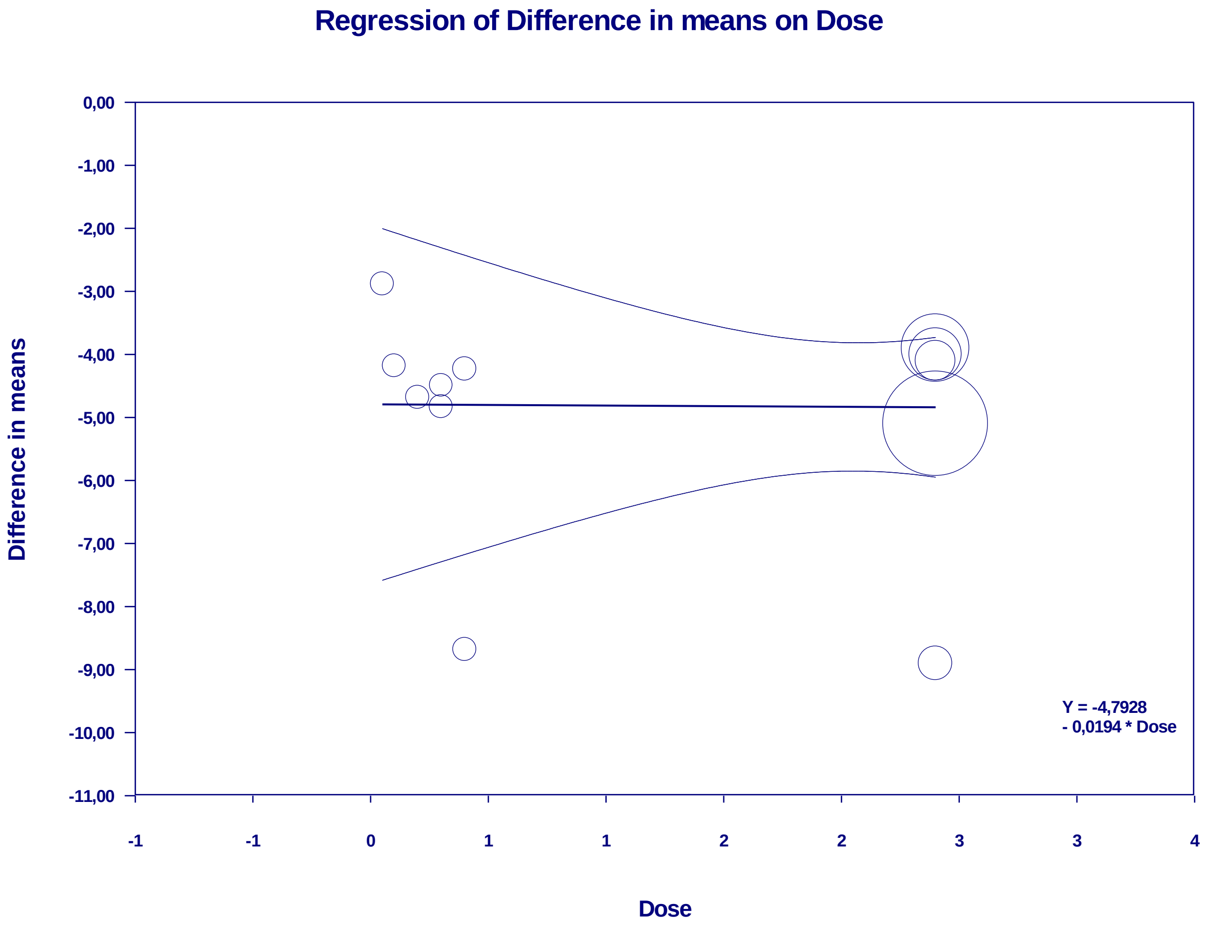

Figure S8: Risk of bias assessment of the included studies.

	Randomization process	Deviations from intended interventions	Missing outcome data	Measurement of the outcome	Selection of the reported result	Overall Bias
O'Neil et al	+	+	+	+	+	+
STEP 1	+	+	+	+	+	+
STEP 3	+	+	+	+	+	+
STEP 4	+	+	+	+	+	+
STEP 5	+	+	+	+	+	+
STEP 8	+	+	+	+	+	+
